# HWP1 Gene Sequence Diversity and Antifungal Susceptibility Patterns in Colombian Vulvovaginal Isolates of the *Candida albicans* Species Complex

**DOI:** 10.1155/ijm/8507361

**Published:** 2025-08-21

**Authors:** Soraya Morales-López, Yeneiris Villero Wolf, Yulibeth Torres, Deyner Lechuga, Luis Caicedo, Guillermo García-Effron

**Affiliations:** ^1^Department of Microbiology, Nancy Florez Garcia Laboratories, Valledupar, Cesar, Colombia; ^2^Department of Microbiology, Universidad Popular del Cesar, Valledupar, Cesar, Colombia; ^3^Department of Mycology and Molecular Diagnostic Laboratory, School of Biochemistry and Biological Sciences, Universidad Nacional del Litoral, Santa Fe de la Vera Cruz, Santa Fe, Argentina

**Keywords:** antifungal susceptibility testing, *Candida africana*, *Candida albicans*, *Candida dubliniensis*, HWP1 gene, vulvovaginal candidiasis

## Abstract

**Background:** *Candida albicans* complex species are the main cause of candidiasis.

**Objectives:** The purpose of this study was to report the prevalence and genetic diversity of *C. albicans* complex using hyphal wall protein 1 (HWP1) gene size polymorphism, as well as the susceptibility patterns to fluconazole and voriconazole.

**Methods:** A total of 170 yeast isolates were obtained from vulvovaginal samples, and phenotypic and proteomic identification was performed.

**Results:** Most clinical isolates were *C. albicans* complex (*n* = 153) followed by *C. glabrata* (*n* = 13), *C. parapsilosis* complex (*n* = 2), and *Pichia kudriavzevii* (*n* = 2). Among *C. albicans* complexes, all isolates were *C. albicans sensu stricto* and 2.61% and 4.58% were resistant to fluconazole and voriconazole, respectively.

**Conclusions:** The presence of different alleles was confirmed, heterozygosity was more common than homozygosity (71.03% vs. 28.97%), and some isolates showed a homozygosis pattern not previously described. Despite these genetic diversities, no specific genotype was linked to azole resistance.

## 1. Introduction

Vulvovaginal candidiasis (VVC) is a common infection that affects millions of women worldwide [1, 2]. In Colombia, prevalence studies estimate that up to 19% of women will experience at least one episode during their lifetime, with recurrent cases becoming an important gynecological concern [3]. In other countries such as Argentina, Brazil, and Ghana*, Candida albicans* remains the prevalent etiological yeast species and VVC has been studied not only in terms of etiological agents but also regarding antifungal susceptibility patterns, highlighting geographic variability [4–7].

The *C. albicans* species complex consists of *C. albicans sensu stricto, Candida dubliniensis, Candida stellatoidea (syn. C. albicans* var. *stellatoidea),* and *Candida africana (syn. C. albicans var. africana),* the latter recognized as an agent of VVC [8–11]. These species and varieties share several phenotypic characteristics, making their identification difficult [12, 13].

In 2008, Romeo and Criseo proposed the first molecular method for discriminating between *C. albicans, C. africana*, and *C. dubliniensis*. This method was based on differences in the hyphal wall protein 1 gene (HWP1) [14]. Subsequently, *C. stellatoidea* was also included using an agarose gel detection method, thereby achieving complete discrimination of the complex. The described method can detect differences in the size of the PCR amplicon as follows: *C. albicans* 941 bp, *C. dubliniensis* 569 bp, *C. africana* 700 bp, and *C. stellatoidea* Type I 800 bp. Widely used, this method constitutes a valuable tool in identifying and discriminating between species or varieties within the complex [15–18].

The HWP1 is an important adhesin expressed in the filamentous form of the yeast after transitioning from blastoconidia to germ tube and hyphae [19]. The HWP1 gene may exhibit genetic polymorphism [20]. Recently, Ngouana et al. described five HWP1 genotypes (named H1 to H5) in *C. albicans* species complex strains. These genotypes consist of the combination of four different HWP1 alleles with varying sizes and were designated as follows: H1 (941 bp in homozygosity), H2 genotype (941 and 1080 bp), H3 (941 + 850 bp), H4 (941+1200 bp), and H5 (850+1080 bp). The authors found no relationship between HWP1 genotypes, antifungal susceptibility patterns, and microsatellite analysis of these strains [21].

This study is aimed at reporting the prevalence and genetic diversity of *C. albicans complex* species using HWP1 gene size polymorphism, as well as the fluconazole and voriconazole susceptibility patterns of *C. albicans* complex strains isolated from vaginal samples in Colombia.

## 2. Materials and Methods

### 2.1. Strains and Identification Procedures

A total of 170 clinical strains isolated from an equal number of women diagnosed with vaginal candidiasis were included in the study. Inclusion criteria included age over 18 years and clinical signs of VVC. The study did not collect available demographic data such as patient age, history of recurrent infections, and geographic origin. All the strains were obtained between 2018 and 2019 and conserved at −86°C. The isolates were streaked onto CHROMagar *Candida* to identify those presumed to belong to the *C. albicans* complex (indicated by green colonies). Subsequently, these presumptive identifications were confirmed using phenotypic [22] and proteomic methods (MALDI-TOF) [23].

The following reference strains were used as controls: (1) *C. albicans* ATCC 90028 and ATCC 10231, (2) *C. africana* CBS 9118, (3) *C. dubliniensis* CBS 7987, and (4) *C. stellatoidea* CBS 1905 and CBS 8190. *C. parapsilosis* ATCC 22019 and *Pichia kudriavzevii* (*C. krusei*) ATCC 6258 were used as control strains for antifungal susceptibility testing.

### 2.2. HWP1 Gene Polymorphism Study for Definitive Speciation

Yeast genomic DNAs were extracted as described previously [24]. For the definitive speciation of the species and varieties of the *C. albicans* complex, the amplification of the HWP1 gene was performed using the PCR described by Romeo and Criseo [14]. PCR products were resolved in a 1.2% agarose gel, and fragments of 941, ∼700, 800, and 569 bp were expected for *C. albicans*, *C. africana, C. stellatoidea*, and *C. dubliniensis*, respectively.

### 2.3. Antifungal Susceptibility Testing

Fluconazole and voriconazole susceptibility were assessed by disk diffusion using Mueller–Hinton agar supplemented with glucose and methylene blue, following the CLSI M44-ED3 method, with 25-*μ*g fluconazole disks (Liofilchem, Italy) and 1-*μ*g voriconazole disks (Liofilchem, Italy) [25]. Results were read at 24 h of incubation at 35°C and interpreted according to the CLSI M27M44SEd3E document [26].

### 2.4. Statistical Analysis

Chi-square test was used for analyzing categorical data to investigate the potential association between azole resistance and the HWP1 polymorphisms. A *p* < 0.05 was considered significant.

## 3. Results

**Figure 1:**
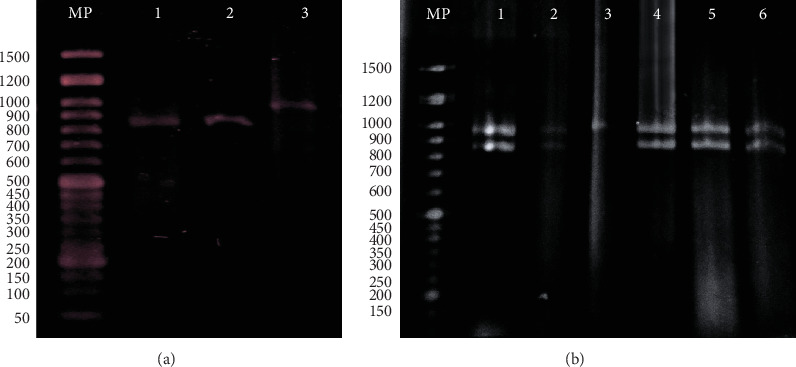
*Hwp1* amplification on gel electrophoresis. MP DNA Ladder. (a) Lanes 1 and 2 *C. albicans* isolates with a single 850 bp and Lane 3, *C. albicans* with a single 940 bp (H1 genotype). (b) Lanes 1, 2, 4, 5, and 6, *C. albicans* isolates with 850 and 941 bp (H3 genotype).

Out of the total 170 isolates, 17 did not show green colonies. Subsequently, they were identified as *Nakaseomyces glabratus* (*Candida glabrata*) (*n* = 13), *Pichia kudriavzevii* (*C. krusei*) (*n* = 2), *Candida parapsilosis* (*n* = 1), and *Candida orthopsilosis* (*n* = 1) by proteomic-based methods (MALDI-TOF). The presumptive identification of the remaining 153 strains as *C. albicans* complex on CHROMagar *Candida* was confirmed using phenotypic methods and by MALDI-TOF. After PCR amplification of the HWP1 genes, the presence of different alleles was confirmed. None of the 153 studied strains showed PCR amplification products of 569, 800, or 700 bp, confirming the absence of *C. dubliniensis*, *C. stellatoidea*, or *C. africana* in our collection and that all strains were *C. albicans*. Among these 153 *C. albicans* strains, only 42 isolates (27.45%) showed a single ~941 bp band in homozygosis, thus considered as belonging to the H1 genotype. On the other hand, 103 (67.33%) strains showed a heterozygous pattern with two bands (850 and 941 bp) (genotype H3). Interestingly, eight strains (5.23%) showed an 850-bp band in homozygosis, a new pattern not previously described (Figure 1).

**Figure 2:**
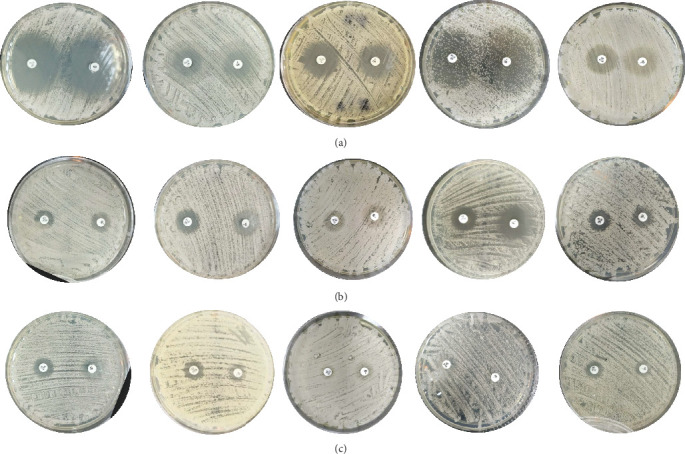
(a) Fluconazole and voriconazole susceptibility strains. (b) Fluconazole SDD and/or voriconazole intermediate strains. (c) Fluconazole- and voriconazole-resistant strains.

In the studied 153 *C. albicans* strains, 139 were found to be susceptible to fluconazole (90.85%), 10 were considered susceptible dose-dependent (6.54%), and 4 were considered resistant to this azole (2.61%). Additionally, 142 were susceptible to voriconazole (S, 92.81%), 4 were considered intermediate (I, 2.61%), and 7 were classified as resistant to this triazole (R, 4.58%) (Figure 2).

When assessing the relationship between azole resistance and polymorphisms in the HWP1 gene, we observed that all but one of the strains resistant to fluconazole and/or voriconazole (*n* = 19) showed heterozygosity in the HWP1 gene (850 and 941 bp). Only one resistant strain was homozygous for the HWP1 gene (941 bp). Despite the presented data, the chi-square test of independence showed that there was no significant association between azole resistance and HWP1 gene polymorphisms, *χ*^2^ (4; 1; *N* = 153) = 4.7949, *p* = 0.309.

## 4. Discussion

Molecular studies focusing on the ITS data sequence demonstrated that *C. africana* is genetically distinct from *C. albicans*, with a sequence homology ranging from 99.3% to 99.8%. Similarly, *C. dubliniensis* exhibited interspecies sequence homology ranging from 91.2% to 94.4% with *C. albicans* [27]. HWP1 gene polymorphisms facilitated the discrimination between them. Using a PCR method based on these differences, three HWP1 alleles with different sizes were obtained: 941, 700, and 569 bp for *C. albicans*, *C. africana*, and *C. dubliniensis,* respectively [14, 28, 29]. This method was employed by several authors, including Gumral et al. (Turkey) [30] who identified all 195 strains as *C. albicans* and detected no *C. africana* or *C. dubliniensis*. Shan et al. (China, 2014) identified 15 isolates out of 1014 presumed *C. albicans* strains as *C. africana*, whereas *C. dubliniensis* was not detected [31]. Similarly, Mucci et al. [32] and Theill et al. [18] (both in Argentina) examined 210 and 287 vaginal specimens, identifying *C. albicans* as the predominant species, with occasional occurrences of *C. dubliniensis* (1.39%) and *C. africana* (0.35%), respectively. Similar findings have been reported in various studies conducted in Nigeria, Senegal, Malaysia, India, Iran, Turkey, and Tunisia, highlighting the scarcity of *C. africana* and *C. dubliniensis* isolates in vaginal samples [33–41] (Table 1). Albaina et al. [42] described atypical *C. dubliniensis* strains isolated in Argentina and Spain that were unable to produce germ tubes and chlamydospores. In these isolates, HWP1 gene amplification gave a 569-bp band that was crucial to identify these isolates as *C. dubliniensis*.

In 2015, a study from Cameroon was the first describing HWP1 polymorphisms in *C. albicans* isolates obtained from HIV-infected patients. In this work, 11 out of 113 strains showed heterozygous HWP1 alleles with different PCR fragment sizes (102 strains with the describe 941-bp fragment, 10 strains with the 941-bp fragment plus an 800-bp fragment, and 1 strain harboring the 941-bp fragment together with a 1080-bp fragment) [20]. Further studies by this group demonstrated the existence of five distinct genotypes (H1 to H5) of the HWP1 gene, including homozygous and heterozygous strains. In this study, 57.52% of the strains were classified as belonging to the H1 genotype (941 bp), 18.58% as H2 genotype (941 and 1080 bp), 16.81% as H3 (941 + 850 bp), 6.19% as H4 (941 + 1200 bp), and 0.9% as H5 (850 + 1080 bp) [21]. Notably, the 800-bp fragment reported by Ngouana et al. in 2015 [20] was not mentioned in the 2016 report of the same group [21]. In the latter report, the 850-bp fragment seems to replace the former size, raising the question of whether the 800-bp and the 850-bp fragments were the same. In other reports, this diversity was also demonstrated [17, 19, 43–46]. Despite these genetic diversities, no specific genotype was associated with azole resistance [21].

Our data show important differences with published reports. Firstly, heterozygosity of HWP1 in *C. albicans* is more common than homozygosity (71.03% vs. 28.97%). Secondly, H3 genotype (941 + 850 bp) was the most prevalent in our Colombian collection of strains, and lastly, eight isolates showed an 850-bp band in homozygosis not previously described.

These differences may be influenced by various factors, including regional antifungal prescription practices, population genetics of *Candida* species, or environmental pressures that shape local fungal ecology.

## Figures and Tables

**Table 1:** Review of the species distribution and *HWP1* gene size polymorphism in *Candida albicans* complex from vaginal candidiasis samples.

**Year**	**n**	**Country**	***C. albicans***	***C. africana***	***C. dubliniensis***	***C. stellatoidea***	***HWP1* fragment size polymorphism (** **n** **)**	**Reference**
2009	163^a^	Italy	139	27	2	0	— 941 bp	[14]
2011	195	Turkey	195	0	0	0	941 bp	[30]
2012	84	Nigeria	82	2	0	0	941 bp	[33]
2012	150	Dakar	109	3	0	0	—	[34]
2013	98	Malaysia	97	0	1	0	941 bp (96) 800 bp (1)	[35]
2014	1014	China	999	15	0	0	941 bp and 839 bp	[31]
2014	128	India	124	4	0	0	941 bp	[36]
2014	115	Cameroon	113	2	0	0	941 (102), 941 + 800 (10), and 941 + 1080 (1)	[21]
2015	114	Iran	109	5	0	0	941 bp	[37]
2016	287	Argentina	(pool)	1	1.39%	0	941 bp	[18]
2017	376	Turkey	370	3	3	0	941 bp	[38]
2017	110	Iran	110	0	0	0	941 bp	[39]
2017	70	Argentina	51	0	3	0	941 bp and 839 bp	[32]
2018	119	Iran	109	10	0	0	941 bp	[8]
2020	53	Tunisia	42	11	0	0	941 bp (49) 941 + 800 bp (15)	[40]
2020	114	Iran	109	3	2	0	941 bp	[41]
2020	100^b^	Iran	97	3	0	0	900 bp	[10]
*2021*	60	Iran	60	0	0	0	850 and 941 bp: 7	[42]
2023	153	Colombia	153	0	0	0	~941 (42), 850-bp band (8), and 850 and 941 bp (103)	Current study

^a^136 strains isolated from vaginal samples and 27 from a culture collection.

^b^70 strains isolated from vaginal candidiasis and 30 from other body sites.

## Data Availability

Data are available on request from the authors.
